# Assessing circuit function in the developing *Xenopus* tadpole: a survey of the behavioral toolkit and underlying neural substrates

**DOI:** 10.3389/fnbeh.2026.1806003

**Published:** 2026-06-18

**Authors:** Merritt Hayes, Andrew Liu, Nicholas Todorovic, Carlos D. Aizenman, Hai-yan He

**Affiliations:** 1 Department of Biology, Georgetown University, Washington, DC, United States; 2 Department of Neuroscience, Brown University, Providence, RI, United States

**Keywords:** associative learning, free-movement pattern Y-maze, innate color preference, multisensory integration, optokinetic and optomotor response, visual avoidance response, schooling behavior, visual acuity

## Abstract

*Xenopus laevis* has long served as an important model for studies of embryonic development and is a cornerstone to our current understanding of cellular and molecular mechanisms underlying neurodevelopment, including the formation and refinement of neural circuits. In recent years, considerable efforts have been made to leverage the great accessibility and amenability of the *Xenopus* system to model neurodevelopmental and neurodegenerative disorders in humans for higher-throughput investigations of genetic and environmental risk factors. Behavioral paradigms are indispensable tools to assess functional output in whole animals. In this review, we summarize a range of behavioral paradigms established in *Xenopus laevis* and *Xenopus tropicalis* tadpoles to evaluate the functional development of the nervous system. We discuss the current understanding of neural substrates that give rise to specific behaviors, and how these paradigms have been used to evaluate the functional ramifications of cellular and molecular abnormalities in neural circuitry. We cover three categories of behavioral paradigms: innate and stable behaviors, experience-dependent and learned behaviors, and social behaviors. Each provides distinct functional readouts of the developing nervous system. We also include tips and suggestions for the implementation of these paradigms and discuss how insights from other aquatic animal models may inform the further development of behavioral paradigms in *Xenopus* tadpoles.

## Introduction

*Xenopus laevis* is a versatile, high-throughput animal model amenable to manipulations at multiple levels. The advantages of the *X. laevis* model include cost-efficiency, high fecundity, and easy access for molecular and cellular manipulation in the externally developing embryos at defined developmental stages (Nieuwkoop and Faber, 1994; Pratt and Khakhalin, 2013; Nenni et al., 2019; Exner and Willsey, 2021). Research on *Xenopus* neurodevelopment has led to fundamental discoveries in various aspects of vertebrate neurodevelopment and regeneration, including neural induction, neural patterning, spinal central pattern generators (CPGs), circuit development, and axon regeneration (Gaze, 1959; Roberts and Clarke, 1982; Harland and Grainger, 2011; Straka and Simmers, 2012; Phipps et al., 2020; Exner and Willsey, 2021). Importantly, the core developmental processes of the *Xenopus* central nervous system are evolutionarily conserved with higher vertebrates, including primates (Lee-Liu et al., 2017; Exner and Willsey, 2021; Willsey et al., 2021). Despite the rapid early development, *X. laevis* tadpoles undergo a relatively protracted period (1–2 weeks) during which the neural circuit is highly plastic and is subjected to experience-dependent refinement and maturation. This provides a critical temporal window for the study of activity-dependent circuit plasticity. For this reason, the *X. laevis* visual system has been widely studied as a model for neural circuit formation and development (Debski and Cline, 2002; Mann et al., 2004; Ruthazer and Aizenman, 2010; Liu et al., 2016; Gao and Shen, 2021). Advancement of *in vivo* calcium imaging (Miraucourt et al., 2016; Li et al., 2022; He et al., 2023; Banerjee et al., 2024), whole-cell patch-clamp recordings (Aizenman and Cline, 2007; Spawn and Aizenman, 2012; James et al., 2015; Truszkowski et al., 2016; Liu H. -H. et al., 2018), and other molecular and biochemical tools, has made headways in elucidating both genetically programmed and experience-dependent developmental processes such as axon guidance, synapse formation, and synaptic maturation within the visual circuit from the cellular to circuit level (Sin et al., 2002; Pratt et al., 2008; Deeg et al., 2009; Ruthazer and Aizenman, 2010; Straka and Simmers, 2012; Pratt and Khakhalin, 2013; Spencer Adams et al., 2014; Koser et al., 2016; He et al., 2022). Additionally, the ease and amenability of tadpoles to intraventricular injection enable highly effective spatiotemporal control for pharmacological manipulation as well as biochemical labeling in the intact nervous system under physiologically relevant conditions (Ahsan et al., 2025; Garrido-Charles et al., 2025).

These characteristics underscore the translational potential of *Xenopus* as a model for neurological disorders and the developmental effects of teratogen exposure (Pratt and Khakhalin, 2013; Gao et al., 2016; Willsey et al., 2024). Historically, the allotetraploid genome and relatively long period to reach sexual maturity (10–12 months) of *X. laevis* presented challenges for genetic tractability and the generation of transgenic lines. To circumvent this, the closely related *Xenopus tropicalis* species was introduced in the late 1990s and has gained traction as a diploid vertebrate model organism for higher-throughput genetic screening and simultaneous manipulation of multiple risk genes as a convergent approach to study neurological disorders (Amaya et al., 1998; Hellsten et al., 2010; Pratt and Khakhalin, 2013; Exner and Willsey, 2021; Willsey et al., 2024; Terni et al., 2026). Recent completion of the genome sequencing and advancement of genome editing toolkits greatly facilitate research using both *X. laevis* and *X. tropicalis* as animal models (for comprehensive reviews on the genetic toolkit development and disease modeling in *Xenopus*, see Tandon et al., 2017; Nenni et al., 2019). Furthermore, the suitability for microinjection at two-cell stage embryos offers a unique and powerful system for molecular genetic manipulations in half of the embryo for within-animal control, as well as circuit tracking of pre- vs. postsynaptic neurons (Li et al., 2026). In recent years, *Xenopus* has been used to model a range of neurological disorders with pharmacological or genetic manipulations, including autism, epilepsy, Fragile X syndrome, Parkinson’s, Huntington’s, multiple sclerosis, and traumatic brain injury (Blonden et al., 2005; Huot et al., 2005, 2012; Pienaar et al., 2010; Gessert et al., 2011; Spawn and Aizenman, 2012; McKeown et al., 2013; Pratt and Khakhalin, 2013; Faulkner et al., 2014; Haremaki et al., 2015; James et al., 2015; Liu and Cline, 2016; Truszkowski et al., 2016; Liu H. -H. et al., 2018; Liu Z. et al., 2018; Mannioui et al., 2018; Sega et al., 2019; Exner and Willsey, 2021; Ismail et al., 2022; Banerjee et al., 2024; Panthi et al., 2024; Park et al., 2024).

Behavioral paradigms provide critical readouts for functional capacity and integrity of neural circuits that may not be fully captured by molecular and histological readouts, and allow for a holistic assessment of the nervous system function under controlled conditions in both healthy and disease states (Henriet et al., 2023). A number of behavioral paradigms have been established in *Xenopus* tadpoles, particularly in *X. laevis* due to its well-characterized development dating back to the 1950s (Gao and Shen, 2021). Behavioral studies in *X. laevis* tadpoles have contributed to seminal findings in the core neuronal properties of spinal CPGs, regenerative processes, and visual system development in vertebrates by connecting anatomical circuit features with functional output (Gaze, 1959; Roberts et al., 1981; Kahn and Roberts, 1982; Roberts and Clarke, 1982; Dale et al., 1986; Dong et al., 2009; Sharples et al., 2014; Shen et al., 2014; Muñoz et al., 2015; Liu et al., 2016; Edwards-Faret et al., 2017; Lee-Liu et al., 2017; Phipps et al., 2020; Knorr et al., 2021; Sindelka et al., 2024). Comparably, very few behavioral paradigms have been established in *X. tropicalis* (Ismail et al., 2022; Terni et al., 2026), and behavioral research in *X. tropicalis* has remained largely restricted to observing phenotypic changes arising from genetic manipulation, such as susceptibility to seizure-like activity and mobility (Sega et al., 2019; Ismail et al., 2022; Park et al., 2024; Terni et al., 2026). In this review, we aim to highlight the key features of behavioral paradigms in *Xenopus* tadpoles that can be generalized from individual studies, identify areas for improvement, and summarize the current understanding of the underlying neural substrates. In doing so, we hope to facilitate the application, implementation, and further development of new paradigms with *Xenopus* tadpoles as a model system in neurodevelopmental and translational studies. Many behavioral paradigms established in tadpoles rely on visual system function to varying degrees, including the optomotor and optokinetic reflexes, color preference, visual avoidance response, visual acuity assay, multisensory integration, aversive conditioning, free-movement pattern Y-maze, and polarized schooling (Katz et al., 1981; Dong et al., 2009; Blackiston et al., 2010; Schwartz et al., 2011; Blackiston and Levin, 2012; Liu et al., 2016; Truszkowski et al., 2016; Hunt et al., 2020; Bruno et al., 2022; Ismail et al., 2022; Thompson and Aizenman, 2023; Udoh et al., 2025). We will primarily focus on premetamorphic *X. laevis* larvae, specifically stages 46–49, during which tadpoles exhibit a range of characteristic behaviors that are related to key developmental events occurring in the visual system. Three main categories of behavioral paradigms will be discussed: innate and stable behaviors, experience-dependent and learned behaviors, and social behaviors.

### Focus on the visual system

**FIGURE 1:**
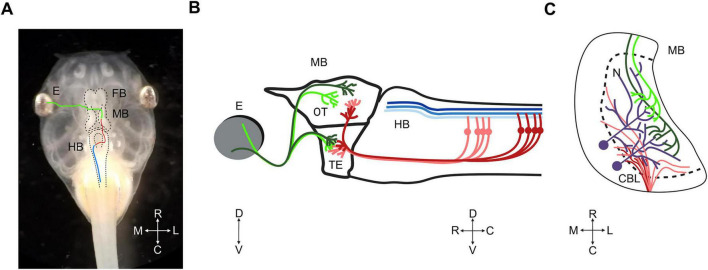
Anatomy of the retinotectal and retinotegmental circuits in *X. laevis* tadpoles. **(A)** Image of stage 47 albino *X. laevis* tadpole. Major sensory projections highlighted: green represents retinal ganglion cell axons carrying visual inputs, red represents hindbrain neuron axons, and blue represents mechanosensory inputs, same in panel **(B,C)**. E, Eye; FB, Forebrain; MB, Midbrain; HB, Hindbrain. **(B)** Sagittal view of the midbrain and hindbrain, illustrating convergence of visual and somatosensory inputs into the tectum and tegmentum neurons. OT, optic tectum; TE, tegmentum. Shades of green, red, and blue demonstrate the topographic organization of the sensory inputs. **(C)** Horizontal view of one side of the optic tectum. CBL, cell body layer; N, neuropil; purple represents tectal neurons. Arrows in panels **(A, C)** denote anatomical axes. R, rostral; C, caudal; M, medial; L, lateral; D, dorsal; V, ventral.

In *X. laevis* tadpoles, nearly all retinal ganglion cell (RGC) axons cross at the optic chiasm and bifurcate, forming the retinotectal circuit through dorsocaudal projections to the neuropil of the optic tectum, and the retinotegmental circuit through ventrocaudal projections to the basal optic nucleus (BON) of the ventrolateral midbrain tegmentum (Figures 1A, B; Sakaguchi and Murphey, 1985; Nieuwkoop and Faber, 1994; Dingwell et al., 2000; Rahman et al., 2020; Udoh et al., 2025; Santos et al., 2022). The optic tectum is located in the dorsal part (roof) of the midbrain and serves as the primary visual processing center in non-mammalian vertebrates. Similar to its mammalian counterpart, the superior colliculus, the central function of the optic tectum is to integrate multisensory inputs to coordinate motor output. In the retinotectal circuit, RGC axons arrive at the contralateral optic tectum at around stage 37/38 and form a coarse retinotopic map in the tectal neuropil (Figure 1C; Sakaguchi and Murphey, 1985; Holt, 1989; Liu et al., 2016). This visual circuit is well-characterized in terms of its developmental properties (Sakaguchi and Murphey, 1985; Holt, 1989; Liu et al., 2016; Santos et al., 2022) and is known to initiate adaptive behaviors in *X. laevis*, such as avoidance responses to approaching visual stimuli (Dong et al., 2009; Lee et al., 2010; Shen et al., 2011, 2014; Spawn and Aizenman, 2012; McKeown et al., 2013, 2024; Khakhalin et al., 2014; Nagel et al., 2015; Gao et al., 2016, 2022; Jang et al., 2016; Liu and Cline, 2016; Liu et al., 2016; Liu H. -H. et al., 2018; Liu Z. et al., 2018; Miraucourt et al., 2016; Truszkowski et al., 2016; Gambrill et al., 2018, 2019; He et al., 2018, 2023; Santos et al., 2018; Liao et al., 2020; Henriet et al., 2023). The tegmentum is located in the floor of the midbrain, ventral to the midbrain ventricle. The direct projection of RGCs to the basal optic nucleus of the tegmentum is known as the accessory optic system (AOS). This highly conserved visual system in vertebrates, including mammals, is known to mediate innate visually guided behaviors that stabilize the visual scene (Gruberg and Grasse, 1984; Simpson, 1984; Masseck and Hoffmann, 2009; Bastakov et al., 2015; Tanaka and Portugues, 2025; Udoh et al., 2025). Both retinotegmental and retinotectal synapses are glutamatergic, although differences in their developmental profiles underlie distinct functional properties, as will be discussed later (Udoh et al., 2025).

Aside from retinal inputs, the tectum and tegmentum also receive hindbrain projections that relay mechanosensory information from the lateral line, vestibular, and trigeminal systems (Deeg et al., 2009; Hiramoto and Cline, 2009; Liu et al., 2016; Udoh et al., 2023). Hindbrain axons project ventrally through the tegmentum, turn dorsally, and terminate on the contralateral tectum in laminae distinct from retinal inputs (Hiramoto and Cline, 2009). Tectal neurons receive mechanosensory inputs from hindbrain neurons on proximal dendrites and visual inputs from RGCs at more distal locations, forming the anatomical basis for the integration of multisensory inputs (Deeg et al., 2009; Hiramoto and Cline, 2009). Retinal and hindbrain innervations of the optic tectum occur at similar developmental stages and are both well-developed by stage 48 (Holt and Harris, 1983; Hiramoto and Cline, 2009). Similar dual hindbrain and retinal innervations have also been demonstrated electrophysiologically in the tegmentum (Udoh et al., 2025), however whether the retinal and hindbrain inputs retain similar anatomical distinction in the tegmentum remains unclear. In developing *X. laevis* tadpoles, all visual inputs are processed monocularly as all RGCs project to the contralateral side of the midbrain (Dingwell et al., 2000). Binocular vision is not achieved until after metamorphosis, when an indirect ipsilateral projection to the tectal lobes is established by around stage 53 via the nucleus isthmi (NI) (Nieuwkoop and Faber, 1994; Udin and Grant, 1999).

## Innate and stable behaviors

### Optomotor response and optokinetic response

**FIGURE 2:**
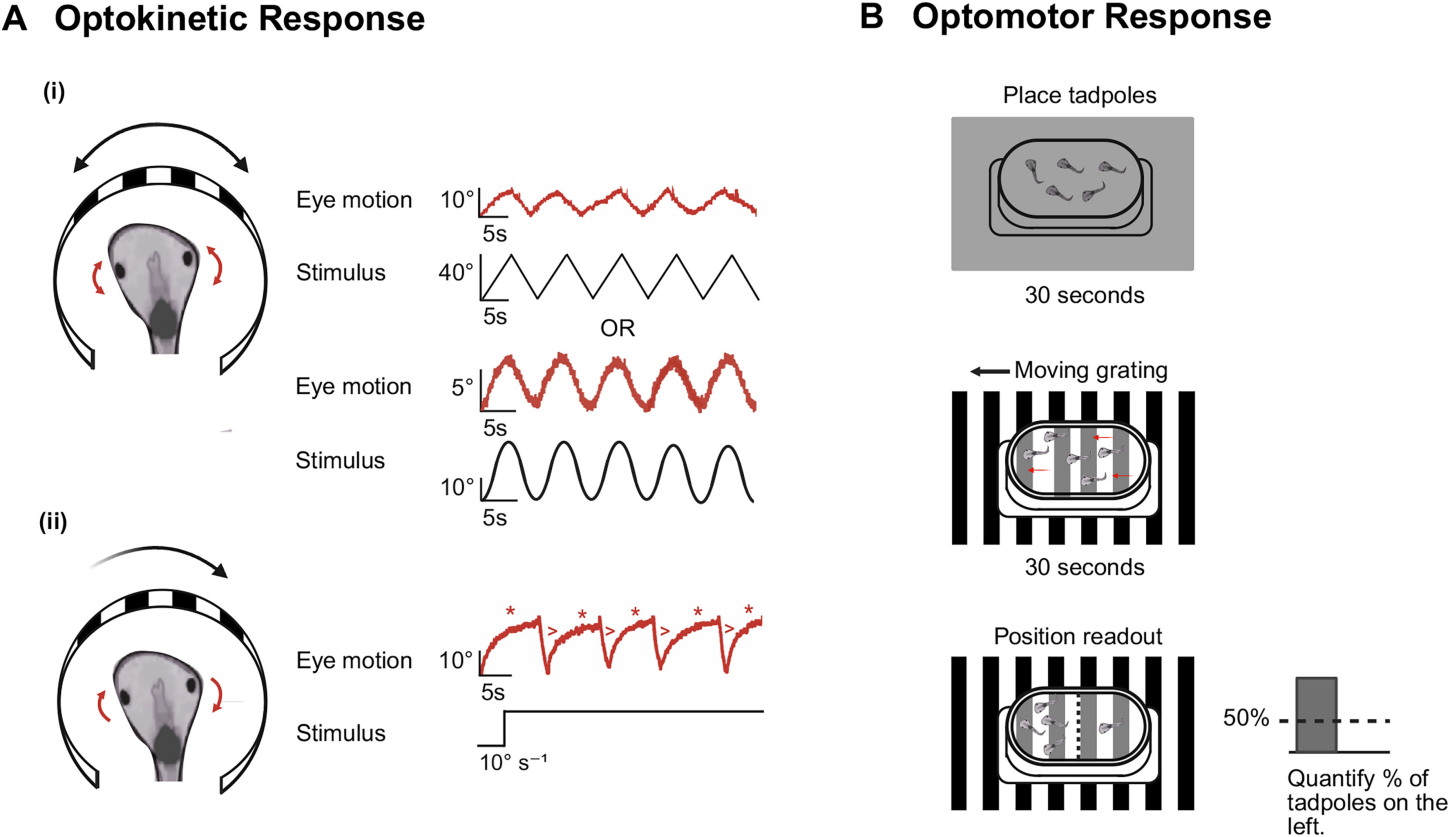
Schematic depiction of two paradigms assessing innate visuomotor behaviors in *X. laevis* tadpoles. **(A)** For OKR evaluation, tadpoles are immobilized on a Petri dish and exposed to a 275° horizontal visual field to evaluate compensatory eye movement and locomotion. Moving stimulus consists of an alternating black and white stripe pattern presented as either back and forth motion (constant velocity: 8° s^– 1^ for 5 s; or sinusoidal: 0.125 Hz with peak velocity ± 10° s^– 1^) or as unidirectional motion (constant velocity: 10° s^– 1^). Representative traces show eye motion over time for the left eye. *, slow phase; >, fast-resetting phase. Adapted from Gravot et al. (2017), Forsthofer and Straka (2023). **(B)** For OMR evaluation, tadpoles are placed in a transparent container and swim in the direction of the moving gratings displayed under the container. Square wave gratings move in the direction perpendicular to the long axis of the container. Representative scoring quantifies the percentage of tadpoles on the left half of the container. Adapted from Dong et al. (2009).

Optic flow describes global movement of the visual scene resulting from relative motion between the observer and the environment. In both insects and animals, optic flow triggers two reflexive movements to maintain visual stability and postural balance: the optokinetic response (OKR) and the optomotor response (OMR) (Roeser and Baier, 2003; Tanaka and Portugues, 2025; Udoh et al., 2025). These reflexes are highly conserved among vertebrates and do not require learning, providing a relatively simple, unbiased assay for hardwired basic visual processing such as direction and contrast sensitivity (Shi et al., 2018). In tadpoles, the OKR and OMR are characterized by movement of either the eyes (OKR, Figure 2A) or whole body (OMR, Figure 2B) in the direction of optic flow (Roeser and Baier, 2003; Udoh et al., 2025). Both OKR and OMR rely on the BON/AOS system through the retinotegmental projections for visual signal processing and are independent of the optic tectum (Pronych et al., 1996; Dong et al., 2009; Knorr et al., 2021; Udoh et al., 2025). The visual information is then integrated with vestibular signals before being sent to motor output centers to instruct the corresponding movement (Udoh et al., 2025). In *X. laevis* tadpoles, retinotegmental synapses remain relatively stable across developmental stages 42–49, and BON neurons retain large receptive fields throughout development into adult frogs, suggesting that the AOS circuit does not undergo significant experience-dependent refinement (Gruberg and Grasse, 1984; Bastakov et al., 2015; Udoh et al., 2025). The reliable AOS synapse and large receptive field of BON neurons likely play a permissive role for the experience-dependent development of other components of the visual system by providing animals a stable visual environment necessary to navigate their surroundings (Simpson, 1984; Exner and Willsey, 2021; Udoh et al., 2025).

To measure OKR, tadpoles are secured to restrict body movement and ensure that only eye movements are permitted. Animals are then presented with horizontally moving images consisting of vertical stripes of alternating color, and monocular eye movements are tracked by a camera (Knorr et al., 2018, 2021). Two types of visual stimulation patterns have been used for OKR evaluation: oscillatory (Figure 2Ai) or unidirectional (Figure 2Aii). When the visual scene moves back and forth in an oscillatory manner (e.g., horizontal motion alternating leftward and rightward, Figure 2Ai), the eye tracks the stimulus with smooth, sinusoidal movements (Gravot et al., 2017; Knorr et al., 2018, 2021; Forsthofer and Straka, 2023). When the visual scene moves steadily in one direction, the eye exhibits nystagmus with a “saw-tooth” pattern consisting of a slow phase of pursuit in the direction of optic flow followed by a fast-reset phase in the opposite direction (Figure 2Aii; Gravot et al., 2017). In addition to alternating patterns of black and white gratings, gratings with alternating colors (e.g., red vs. blue) can also invoke OKR responses in *X. laevis* tadpoles (Knorr et al., 2018, 2021). Functional OKR is observed as early as stage 44 in *X. laevis* tadpoles (Dong et al., 2009; Knorr et al., 2018, 2021; Bacqué-Cazenave et al., 2022; Forsthofer and Straka, 2023). Interestingly, recent findings suggest that in older tadpoles (stage 50–56), the optokinetic response undergoes bidirectional adaptive plasticity to maintain the magnitude of optokinetic eye movements within a functional range (Forsthofer and Straka, 2023). The full expression of this behavioral plasticity coincides with the maturation of cerebellar circuits, suggesting the recruitment of cerebellar circuits in addition to the AOS over development (Forsthofer and Straka, 2023).

To observe the optomotor response, tadpoles are placed in an open-field container and exposed to drifting gratings of alternating contrast projected underneath the container, and their corresponding orienting movements are measured (Pronych et al., 1996; Roeser and Baier, 2003; Dong et al., 2009). The percentage of tadpoles that end up on the side corresponding to the direction of the drift after 30 s is quantified, which is typically well above 50% as tadpoles tend to swim in the direction of the moving stripes (Dong et al., 2009). Consistent with electrophysiological data demonstrating relatively stable AOS synapses (Udoh et al., 2025), OMR in *X. laevis* tadpoles appears to be stable across stages 44–49 (Dong et al., 2009). The behavior is unaffected by the ablation of the optic tectum, indicating that the retinotectal circuit is not required for OMR in tadpoles (Dong et al., 2009). The consistent OMR performance across development makes this paradigm an invaluable assay for general health and basic visuomotor function (Roeser and Baier, 2003; Dong et al., 2009; Portugues and Engert, 2009; Shen et al., 2011; McKeown et al., 2013; He et al., 2018, 2023). Individual tadpoles can be screened by their tendency to turn toward and follow the moving gratings as a criterion for inclusion in other subsequent visually-guided behavioral experiments (McKeown et al., 2013; He et al., 2018, 2023).

Supporting the notion that the AOS is evolutionarily conserved, optogenetic studies of the AOS in larval zebrafish and the homologous lobula plate in *Drosophila* flies indicate that these brain regions are causally implicated in detecting optic flow and generating OKR and OMR behaviors (Tanaka and Portugues, 2025). The AOS in vertebrates, including amphibians, includes a pretectal nucleus (Nucleus of the optic Tract, NOT) and the BON in the tegmentum (Masseck and Hoffmann, 2009). In zebrafish, the AOS consists primarily of the pretectum, rather than the tegmentum (Matsuda and Kubo, 2021). In tadpoles, the BON was recently characterized (Udoh et al., 2025), but the contribution of the pretectum to OMR and OKR behaviors remains unclear. Electrophysiological studies in *Rana temporaria* suggest that retinal inputs project directly to two discrete midbrain nuclei, the pretectum and the BON, to produce gaze stabilization behaviors (Cochran et al., 1984). The pretectal region is predominantly sensitive to movement in the temporal-to-nasal direction (hence horizontal optokinetic stimulation), while the basal optic region contains neurons that preferentially respond to vertical stimulation but also have some sensitivity in the nasal-to-temporal direction (Cochran et al., 1984). Furthermore, in another frog species, *Rana esculenta*, lesions to the basal optic region fibers eliminated vertical optokinetic behavior while leaving horizontal optokinetic behavior intact (Lázár, 1983). The functional specificity of these different circuit components in tadpoles remains to be fully elucidated.

### Innate color preference

Tadpoles, like many other animals, exhibit an innate color preference in which they exhibit phototaxis toward favorable wavelengths of light (Moriya et al., 1996; Hunt et al., 2020; Bruno et al., 2022; Terni et al., 2026). Both *X. laevis* and *X. tropicalis* tadpoles show a preference for mid-spectrum wavelength (green) color instead of longer wavelength (red), although their preference for shorter wavelength (blue) seems to differ (Hunt et al., 2020; Terni et al., 2026). This preference does not require visual experience, as animals reared exclusively in the dark throughout development displayed normal phototaxis toward green backgrounds (Hunt et al., 2020). Ablation experiments revealed that, similar to the OMR and OKR behaviors, the innate color preference requires the tegmentum, but not the optic tectum (Hunt et al., 2020). At the peripheral end, the retina of *X. laevis* tadpoles contains five distinct types of photoreceptors, including two different rods and three different cones (Zhang et al., 1994; Wilhelm and Gábriel, 1999; Röhlich and Szél, 2000). The retina of *X. laevis* tadpoles matures by stage 43/44 as indicated by the expression of the classical opsin and becomes fully functional (Man et al., 2023).

**FIGURE 3:**
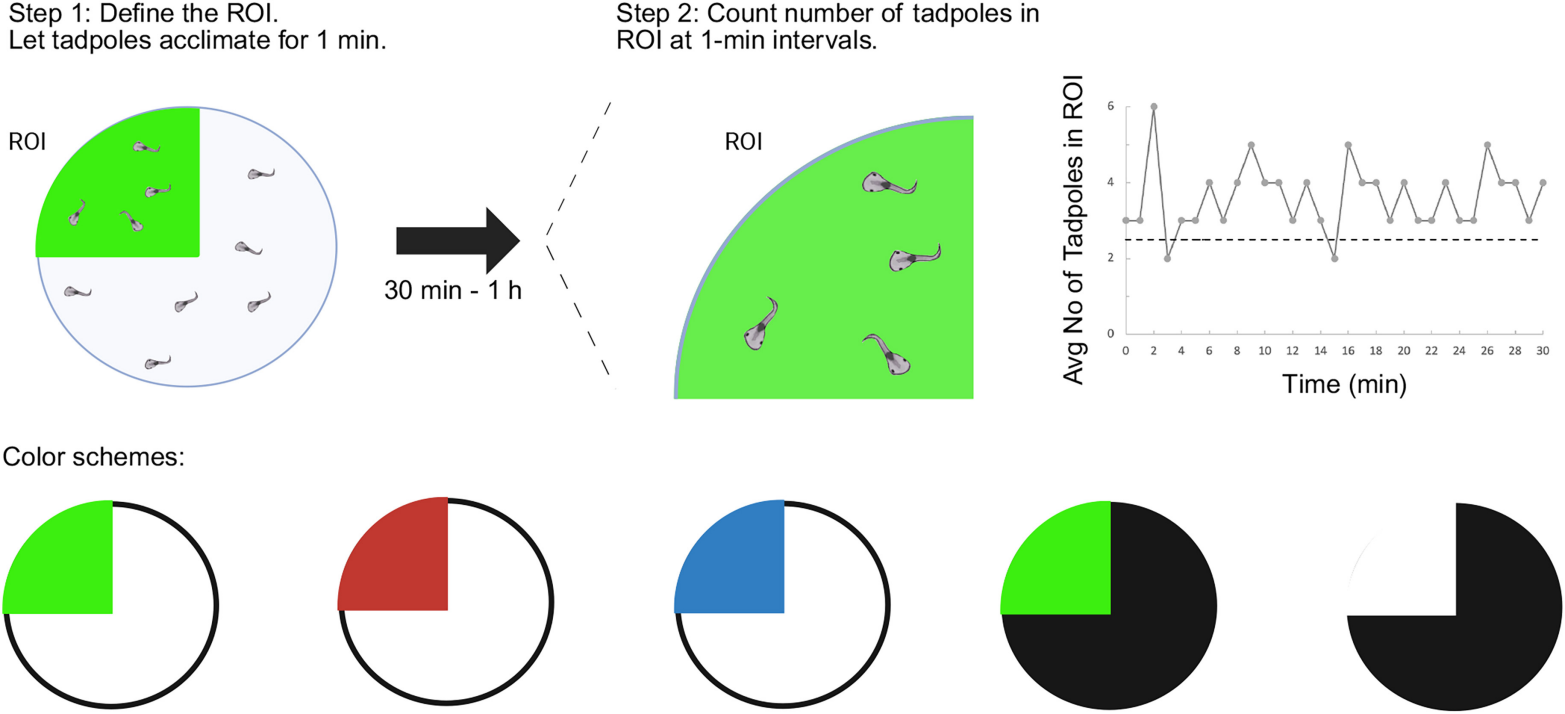
Innate color preference assay in *X. laevis* tadpoles. Experimental design. The ROI is defined as the quarter of the dish with a different color background. Tadpoles are allowed to acclimate for 1 min and then are videotaped as they swim freely for either 30 min or 1 h. The number of tadpoles in the ROI is counted every minute. The solid line represents the mean number of tadpoles in the ROI at each 1-min time point (averaged across trials). The dashed line represents the expected mean under no-preference conditions (e.g., all white). Examples of ROIs defined by contrasting color pairs. Adapted from Hunt et al. (2020).

The innate color preference of tadpoles is quantified by measuring color attraction in freely swimming animals, as detailed in a protocol by Udoh et al. (2023). In short, a circular dish is divided into four quadrants, with one quadrant—the region of interest (ROI)—displaying a different color from the rest (Figure 3). For each experimental session, 10 tadpoles are placed into the dish and allowed to swim freely while a camera takes time-lapse images of the animal distribution. The number of tadpoles in the ROI is counted every minute. Tadpoles are considered to be in the ROI only if both eyes, or one eye and the majority of the head, are in the ROI quadrant (Hunt et al., 2020). The test is performed at around the same time every day to minimize any circadian-dependent influence.

In addition to color preference, both *X. laevis* and *X. tropicalis* tadpoles exhibit a circadian-dependent preference for light over dark environments (Moriya et al., 1996; Bruno et al., 2022; Terni et al., 2026). When stage 48/49 tadpoles are reared on a 12:12 h light/dark schedule, they show a strong preference for white over black background during daytime and show no preference for either condition during nighttime (Bruno et al., 2022). This circadian-dependent preference for light is modulated by the endogenous serotonin level and is accentuated by treatment with selective serotonin reuptake inhibitors (SSRIs) and weakened by inhibiting serotonin release (Hunt et al., 2020; Bruno et al., 2022). Such positive correlation between serotonergic signaling and light preference has also been reported in other animal species, including *Drosophila* (Rodriguez Moncalvo and Campos, 2009), zebrafish (Cheng et al., 2016), crabs (McPhee and Wilkens, 1989), and crayfish (Shiratori et al., 2017). Interestingly, the preference for light displayed by *X. laevis* tadpoles declines over development during the premetamorphic and metamorphic stages, although the underlying mechanism is unclear (Moriya et al., 1996; Bruno et al., 2022).

Notably, the above studies used wild-type (pigmented) tadpoles. Interestingly, a recent study revealed that albino *X. laevis* tadpoles prefer dark backgrounds when compared to wild-type tadpoles (Adebogun et al., 2023). This preference persists even when tadpoles had their pineal glands removed or their optic nerve severed, indicating neither circuit is solely responsible for this phototaxis (Adebogun et al., 2023). Pineal photoreceptors have been previously described to detect luminance and wavelength changes and initiate swimming in response to changes in ambient light intensity, independent of visual inputs (Roberts, 1978; Blackiston and Levin, 2013a; Li et al., 2014; Butler et al., 2022). In *X. laevis* tadpoles, the pineal complex is functional by stage 43 and regulates circadian oscillations by secreting melatonin in the dark and modulates skin pigmentation independent of visual inputs (Bertolesi et al., 2022). Further experiments involving ablations of the optic nerve and the pineal gland, as well as inputs from photoreceptors on the skin, may elucidate the sensory inputs that are required for phototaxis in tadpoles (Bertolesi et al., 2022; Adebogun et al., 2023). On the other hand, such differences in the innate preference for light and dark should be taken into account when designing experiments for different strains of tadpoles.

## Experience-dependent and learned behaviors

### Visual avoidance response

The visual avoidance response (VAR) quantifies visuomotor integration in *X. laevis* tadpoles by assessing their behavioral response to an approaching visual stimulus based on changes in swimming speed and trajectory (Dong et al., 2009; Lee et al., 2010; Spawn and Aizenman, 2012; McKeown et al., 2013, 2024; Khakhalin et al., 2014; Shen et al., 2014; Nagel et al., 2015; Gao et al., 2016, 2022; Jang et al., 2016; Liu and Cline, 2016; Liu et al., 2016; Liu H. -H. et al., 2018; Liu Z. et al., 2018; Miraucourt et al., 2016; Truszkowski et al., 2016; Gambrill et al., 2018, 2019; He et al., 2018, 2023; Santos et al., 2018; Liao et al., 2020; Khakhalin, 2021; Henriet et al., 2023).

#### Variations of the visual avoidance response paradigm

**FIGURE 4:**
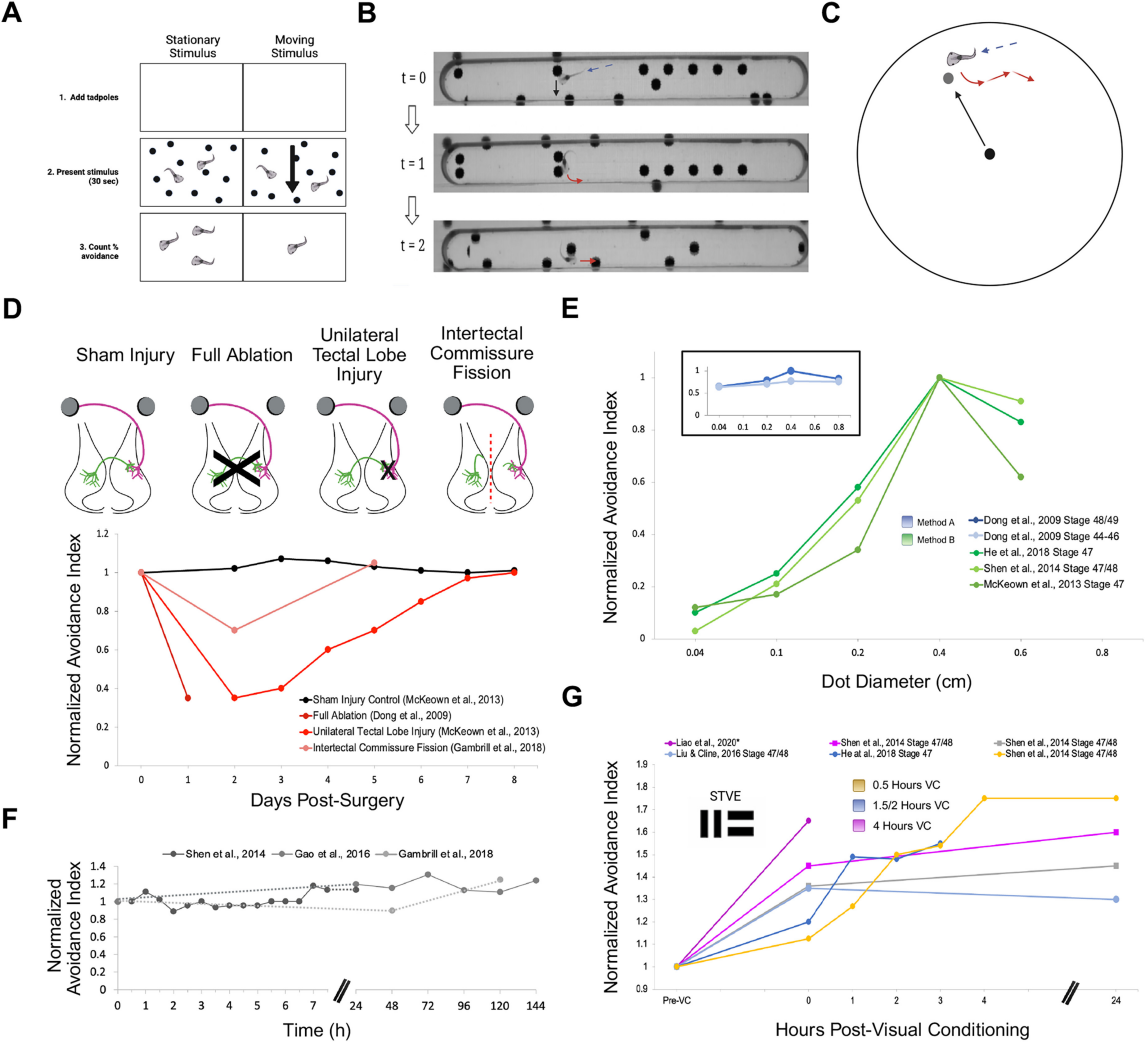
Summary of studies using the visual avoidance response (VAR) paradigm to evaluate retinotectal circuit development and function in *X. laevis* tadpoles. **(A–C)** Different variations of the VAR paradigm. **(A)** Batch analysis assay. Adapted from Dong et al. (2009). **(B)** Individual trajectory-based assay with single-lane analysis. Tadpoles are encouraged to swim horizontally in individual lanes to increase the number of encounters with moving dots. **(C)** Collision avoidance assay. Adapted from Nagel et al. (2015). **(D)** Ablation or injury of the optic tectum significantly disrupts the avoidance response. Adapted from Gambrill et al. (2018). **(E)** The VAR responses of tadpoles are tuned to a specific stimulus size of 0.4 cm diameter circular dots. Inset: The tuning curve sharpens over development. **(F)** Repetitive testing does not cause habituation of VAR. **(G)** Visual conditioning with STVE significantly improves VAR performance. Longer conditioning induces higher visual avoidance responses when tested immediately after conditioning, but the effects at 24 h are comparable across conditioning durations. *, stage of tadpole not specified. Based on Dong et al. (2009), McKeown et al. (2013), Gambrill et al. (2018), Shen et al. (2014), Liao et al. (2020), Liu and Cline (2016), and He et al. (2018).

Originally introduced by Dong and colleagues in 2009, the VAR paradigm has been adapted with three variations: (1) Batch avoidance assay (Figure 4A; Dong et al., 2009; Lee et al., 2010; Spawn and Aizenman, 2012). A group of four tadpoles is allowed to swim freely in an open-field container while moving dots are presented on the bottom of one half of the container, and stationary dots are presented on the other half. After 30 s, the number of tadpoles on the stationary side is counted to calculate the percentage of avoidan**c**e. (2) Trajectory-based assays (Figure 4B; Shen et al., 2011, 2014; McKeown et al., 2013, 2024; Gao et al., 2016, 2022; Liu and Cline, 2016; Liu et al., 2016; Liu H. -H. et al., 2018; Liu Z. et al., 2018; Miraucourt et al., 2016; Gambrill et al., 2018, 2019; He et al., 2018, 2023; Liao et al., 2020). Individual tadpoles are placed into a container (one tadpole each time) or a container with separated lanes and allowed to acclimate. Random moving dots are projected onto the bottom of the container for 60 s, and tadpole behavior is videotaped. Videos are analyzed *post hoc* on a frame-by-frame basis. An encounter is scored when a moving dot passes in front of the tadpole at a perpendicular angle (∼90 ± 15°), and an avoidance response is scored when tadpoles exhibit an abrupt change in swimming trajectory immediately following the encounter (usually within 0.5 s). The Avoidance Index quantifies the proportion of avoidance responses over the first 10 encounters. Tadpoles that do not reach the minimum of 10 encounters during the testing period are excluded from data analysis. (3) Collision avoidance assay (Figure 4C; Khakhalin et al., 2014; Nagel et al., 2015; Jang et al., 2016; Truszkowski et al., 2016; Santos et al., 2018; Khakhalin, 2021; Henriet et al., 2023). An individual tadpole is allowed to swim freely in a circular dish (typically 8.5 cm diameter). Every 30 s, a moving dot is launched by an experimenter or a computerized program from the center of the dish directly at the tadpole to intercept the tadpole’s swimming trajectory. Up to 20 dots are presented per tadpole (Khakhalin, 2021). Similar to the trajectory-based assay, an Avoidance Index is calculated by the proportion of avoidance responses over total encounters.

#### Characterization and applications of the VAR paradigm

The visual avoidance response is a tectally mediated behavior that is significantly disrupted when the optic tectum is injured or removed (Figure 4D; Dong et al., 2009; McKeown et al., 2013; Gambrill et al., 2018). Following partial injuries, the response recovers alongside the recovery of the tectal circuits (McKeown et al., 2013; Gambrill et al., 2018). The VAR performance of tadpoles improves across developmental stages 46–48/49, coinciding with the maturation of the retinotectal circuit (Figure 4E; Dong et al., 2009; McKeown et al., 2013; Shen et al., 2014; Liu and Cline, 2016; He et al., 2018). The refinement of receptive field properties in the tectal neurons during this period is evidenced by the sharpening of the tuning curve across developmental stages 44–49 for the preferred stimulus size of about 0.4 cm dots, which is notably similar in size to the tadpole head (Figure 4E; Dong et al., 2009). Importantly, the avoidance response does not habituate with repeated measurement (Figure 4F; Shen et al., 2014; Gao et al., 2016; Gambrill et al., 2018), making it feasible to perform within-batch longitudinal experiments with repeated measurement designs.

As a behavioral readout for visuomotor integration, the VAR paradigm has been used to evaluate the functional development of tectal circuits (Dong et al., 2009; Lee et al., 2010; Shen et al., 2011; Spawn and Aizenman, 2012; McKeown et al., 2013, 2024; Khakhalin et al., 2014; Nagel et al., 2015; Gao et al., 2016, 2022; Jang et al., 2016; Liu and Cline, 2016; Miraucourt et al., 2016; Truszkowski et al., 2016; Liu H. -H. et al., 2018; Santos et al., 2018; Khakhalin, 2021; Henriet et al., 2023). This readout is particularly valuable in assessing the functional implications of developmental deficits without dysfunction in locomotive or innate behaviors. For example, manipulating the expression of Down syndrome cell adhesion molecule (DSCAM) in the retinotectal circuit alters RGC axon branching and tectal neuron dendrite growth and is sufficient to impair performance on the VAR paradigm while leaving general locomotor activity unaffected (Santos et al., 2018).

In addition to testing the functional output of the retinotectal circuit, the VAR is a robust assay of experience-dependent behavioral plasticity (Hiramoto and Cline, 2009; Shen et al., 2014; Liu and Cline, 2016; Gambrill et al., 2018, 2019; He et al., 2018, 2023; Liu H. -H. et al., 2018; Liao et al., 2020). The retinotectal circuit undergoes a period of highly dynamic experience-dependent synaptic development and refinement between stages 44–48 before entering a relatively stable phase at stage 49 (Sakaguchi and Murphey, 1985; Akerman and Cline, 2006; Pratt et al., 2008; Liu et al., 2016). During this period, the retinotopic map formed by RGC axons on tectal neurons is actively refined by incoming visual experience through activity-dependent plasticity mechanisms (Cline and Constantine-Paton, 1990; Wu et al., 1996; Zhang et al., 2000; Engert et al., 2002; Sin et al., 2002; Ruthazer et al., 2003; Haas et al., 2006; Mu and Poo, 2006; Aizenman and Cline, 2007; Chiu et al., 2008; Pratt et al., 2008; Dunfield and Haas, 2009; Lim et al., 2010; Deeg and Aizenman, 2011; Xu et al., 2011; Chen et al., 2012; Podgorski et al., 2012; Shen et al., 2014; He et al., 2016, 2018; Liu et al., 2016; Truszkowski et al., 2017; Gore et al., 2021; Li et al., 2022; Kutsarova et al., 2023; Huang et al., 2024; Udoh et al., 2025). Exposing animals to a short-term enhanced visual experience (STVE) accelerates the experience-dependent maturation of the retinotectal circuit that otherwise occurs naturally over development (Sin et al., 2002; Aizenman et al., 2003; Haas et al., 2006; Hiramoto and Cline, 2014; Shen et al., 2014; He et al., 2016, 2018; Liu and Cline, 2016; Gambrill et al., 2018, 2019; Liu H. -H. et al., 2018; Liao et al., 2020). STVE typically presents full-field sinusoidal or square wave gratings drifting in 8 directions with 45° intervals in a pseudo-random order. Each 10 min session consists of 5 min of drifting gratings followed by 5 min of equal-luminance gray screen (Hiramoto and Cline, 2009; Shen et al., 2014; Gambrill et al., 2018, 2019; He et al., 2018, 2023). Visual conditioning ranges from 3 to 24 total sessions (30 min to 4 h). Importantly, 30 min of STVE is sufficient to induce long-lasting improvement of VAR performance that endures for at least 24 h (Figure 4G; Shen et al., 2014). STVE-induced structural and functional plasticity in tectal neurons and subsequent improvement of VAR performance is dependent on canonical activity-dependent plasticity mechanisms, such as *N*-methyl-D-aspartate receptor (NMDAR) signaling, activation of calcium/calmodulin-dependent protein kinase II (CaMKII), and protein synthesis (Shen et al., 2014). The STVE-VAR paradigm provides a pivotal readout at the circuit level to distinguish mechanisms specifically relevant to experience-dependent plasticity from those affecting basal circuit function in general. For example, inhibiting the degradation of activity-induced nascent proteins mediated by neuronal membrane proteasomes (NMPs) was found to significantly impair VAR performance in tadpoles following STVE but had no effect in the absence of STVE, allowing the researchers to pinpoint the function of NMPs to be specifically required for experience-dependent plasticity mechanisms (He et al., 2023).

#### Methodological considerations for optimization

Each VAR paradigm variation offers distinct advantages and limitations. The batch avoidance assay is relatively quick and amenable to high-throughput screening but does not capture individual tadpole responses to moving stimuli, potentially reducing sensitivity for detecting subtle circuit-level phenotypes (Dong et al., 2009). The collision avoidance assay ensures that the moving stimulus intercepts each tadpole’s trajectory and can achieve avoidance rates of 70%–80% in controls (Khakhalin, 2021). The collision avoidance assay uses Ethovision to track the animal and the dots semiautomatically (Khakhalin, 2021). In addition to the visual avoidance index, other measurements such as collision angle and response, perceived rate of looming, and time delay of the avoidance can also be measured, which allows more nuanced assessment of individual responses and the detection of behavioral differences across stimuli of varying size and velocity (Khakhalin et al., 2014; James et al., 2015). For example, small, fast stimuli (0.28 cm, 2.9 cm/s) elicit a stereotyped C-shaped body bend followed by rapid swimming, whereas large, slow stimuli (0.56 cm, 1.4 cm/s) tend to elicit a more deliberate avoidance maneuver (Khakhalin et al., 2014; James et al., 2015). Neither the batch assay nor the collision avoidance assay has been used in studies involving experience-dependent plasticity, but the collision avoidance assay has been used to model autism phenotypes and the functional consequences of demyelination and remyelination (James et al., 2015; Henriet et al., 2023). The more limited application of the collision avoidance assay compared to the individual trajectory-based assays may reflect its longer experimental design, including testing times of up to 15 min per tadpole (Khakhalin, 2021), which may constrain batch sizes and limit high-throughput studies.

The individual trajectory-based assay is the most widely used and has been applied across studies of visual system development, injury, regeneration, and experience-dependent plasticity (Shen et al., 2011, 2014; McKeown et al., 2013; Gao et al., 2016, 2022; Liu and Cline, 2016; Miraucourt et al., 2016; Gambrill et al., 2018, 2019; He et al., 2018, 2023; Liu H. -H. et al., 2018; Liu Z. et al., 2018; Liao et al., 2020). This variation allows direct evaluation of individual tadpole behavior upon stimulus encounter and can potentially support higher throughput studies via multi-lane recording of multiple tadpoles per trial (He et al., 2023; McKeown et al., 2024). However, data analysis in this variation remains the most cumbersome, as videos are typically scored manually on a frame-by-frame basis. Usually, a double-blinded experimental design and cross-validation of the scoring from multiple experimenters are employed to circumvent the subjectivity caveat. Excitingly, an open-sourced automated tracking program X-Tracker was published recently, which allows automatic scoring of VAR performance in recorded videos with human-level accuracy in a fraction of the time (McKeown et al., 2024). To automatically distinguish and track the animal from the visual stimuli background, it requires fairly strict lighting conditions, which may pose obstacles for the implementation in different lab settings. Other machine learning algorithms, such as SLEAP, have also been developed and successfully applied in behavioral analyses in rodents, zebrafish, and other animal models (Franco-Restrepo et al., 2019). Successful implementation of these automatic analysis programs in the VAR paradigm will significantly reduce the data analysis time, enable high-throughput processing of larger datasets, and increase the reliability and reproducibility of behavioral quantification for future experiments.

For labs implementing the VAR paradigm, a few factors should be considered to optimize the protocol. (1) Tadpoles can be prescreened with the OMR to exclude animals with deficits in tectally-independent visuomotor circuit function (Dong et al., 2009; Portugues and Engert, 2009; Shen et al., 2011; McKeown et al., 2013; He et al., 2018, 2023; Hunt et al., 2020). (2) The avoidance response is empirically found to be most robust when experiments are performed in the morning (Khakhalin, 2021; Henriet et al., 2023). This could be due to higher locomotive activities generally observed in tadpoles in the morning, or there may be circadian factors involved. (3) Tadpoles tend to be less active when satiated. Hence, they should be fed the night before the experiment, but not in the hours immediately preceding the experiment, to ensure sufficient activity levels for encounters with visual stimuli (Khakhalin, 2021; Henriet et al., 2023). (4) It is important to isolate the setup from other visual scenes, such as in an enclosed chamber, so that the tadpoles are not exposed to other visual inputs (Henriet et al., 2023). (5) Maintaining a low level of diffuse ambient light (e.g., a uniform gray background) in between trials also improves performance (unpublished observation). (6) Other stimulus parameters to consider: a dot diameter of 0.4 cm typically elicits the most robust avoidance response (Dong et al., 2009; Shen et al., 2014). Velocities of moving dots used range from 2 cm/s (Dong et al., 2009) to 3–5 cm/s (Khakhalin, 2021). A dot luminance of 57 cd/m^2^ yields robust avoidance behavior, while decreasing luminance progressively worsens this behavior (Dong et al., 2009). Thus, it is important to calibrate each new experimental setup with a series of dot sizes, velocities, and luminance to account for individual differences between experimental setups and optimize behavioral responses to each setup.

### Visual acuity assay

**FIGURE 5:**
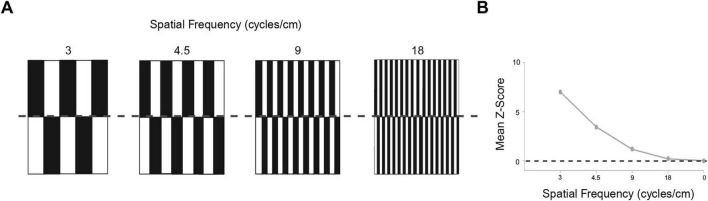
Visual acuity assay in *X. laevis* tadpoles. **(A)** Tadpoles are exposed to sine wave gratings counterphasing at 4 Hz and 80% contrast across a range of spatial frequencies (3, 4.5, 9, and 18 cycles/cm). **(B)** Mean Z-scores from individual tadpoles calculated at each spatial frequency, which quantifies the standard deviations of the mean stimulus-evoked response from the mean response recorded in the absence of any stimulus. Adapted from Thompson and Aizenman (2023).

Visual acuity measures the ability of the visual system to resolve fine spatial details and can serve as a sensitive readout of visual circuit function as well as maturation. In tadpoles, visual acuity improves as the retinotectal circuit matures and the receptive field size of tectal neurons becomes smaller across developmental stages 46–49 (Schwartz et al., 2011; Thompson and Aizenman, 2023). A behavioral paradigm has been established in *X. laevis* tadpoles to assess visual acuity by evaluating escaping behavior triggered by counterphasing gratings (Schwartz et al., 2011; Truszkowski et al., 2017; Thompson and Aizenman, 2023). In this paradigm, individual tadpoles are placed into a small dish and presented with counterphasing sine wave gratings of varying spatial frequencies on the bottom of the container in a block-randomized order (Figure 5A; Thompson and Aizenman, 2023). A baseline period (e.g., 90 s) of static grating is presented before each testing block, in which the light and dark bars of the grating reverse position at 4 Hz for 4 s, followed by a 30 s interstimulus interval. Tadpoles respond to the counter-phasing of gratings with an erratic swimming behavior characterized by increased rates of trajectory acceleration and frequent changes in swimming direction (Schwartz et al., 2011). The behavioral response can be measured either by the probability of evoking a response or by the z-score of changes in velocity, and decreases as spatial frequency increases (Figure 5B; Schwartz et al., 2011; Thompson and Aizenman, 2023). This relationship was corroborated by electrophysiological recordings showing a comparable decrease in the amplitude of visually-evoked synaptic responses in tectal neurons with increasing spatial frequency (Schwartz et al., 2011). The visual acuity of each animal can then be extrapolated by the behavioral response to different spatial frequencies (Schwartz et al., 2011).

Like the visual avoidance response, experience-dependent plasticity in the retinotectal circuit improves visual acuity (Schwartz et al., 2011). Brief periods (20 min) of enhanced visual conditioning in stage 47 tadpoles trigger an activity-dependent upregulation of brain-derived neurotrophic factor (BDNF), which promotes bidirectional synaptic plasticity at retinotectal synapses and increases visual acuity that can be detected both electrophysiologically and behaviorally (Schwartz et al., 2011). Additionally, disruption of the expression of voltage-gated sodium channels during developmental stages 44–46 is sufficient to produce lasting deficits in visual acuity at stage 49, demonstrating that intrinsic excitability plays a crucial role in normal visual system development in tadpoles (Thompson and Aizenman, 2023). Together, these findings highlight the utility of the visual acuity assay in evaluating visual function and detecting the functional consequences of disruptions to the retinotectal circuit in tadpoles.

Factors to consider for implementation of this paradigm: (1) A range of spatial frequencies that span the dynamic range of the response should be tested. (2) Optimize the interstimulus interval with control animals. Stimuli presented too close together will lead to habituation, and too far apart will lead to very long experiments with fewer presentations. We find that 20–30 s between stimuli minimizes habituation, however this may vary with different experimental settings and should be systematically calibrated for every new setup with a wide range of intervals tested. The shortest interval that consistently induces a robust response should be used.

### Multisensory integration

As previously described, in addition to visual processing, the optic tectum is the primary multisensory processing center in the tadpole (Deeg et al., 2009; Hiramoto and Cline, 2009; Truszkowski et al., 2017). Multisensory integration (MSI) is the process by which neural responses to simultaneous inputs from multiple sensory modalities are integrated to produce a more reliable motor response than from unimodal inputs (Hiramoto and Cline, 2009), and can be evaluated by the modulation of response to inputs from one modality by inputs from another modality (Felch et al., 2016). Previous studies of MSI in other animal models revealed that the degree of crossmodal modulation is governed by several factors, including the relative strength of each unimodal input, the spatial congruence of their receptive fields, and the temporal interval separating the two stimuli (Wallace et al., 1998, 2006). In tadpoles, the maturation of MSI in tectal neurons between developmental stages 44–46 and 48–49 is manifested by a decreased integration temporal window (i.e., interstimulus intervals, ISI) and sharpened tuning for ISI, a process that correlates with the developmental increase in recurrent inhibition within the tectal circuit (Felch et al., 2016). As such, the recently developed multisensory integration behavioral paradigm in *X. laevis* tadpoles offers a robust functional assay for circuit development and function of the optic tectum in tadpoles (Truszkowski et al., 2017; Thompson and Aizenman, 2023).

**FIGURE 6:**
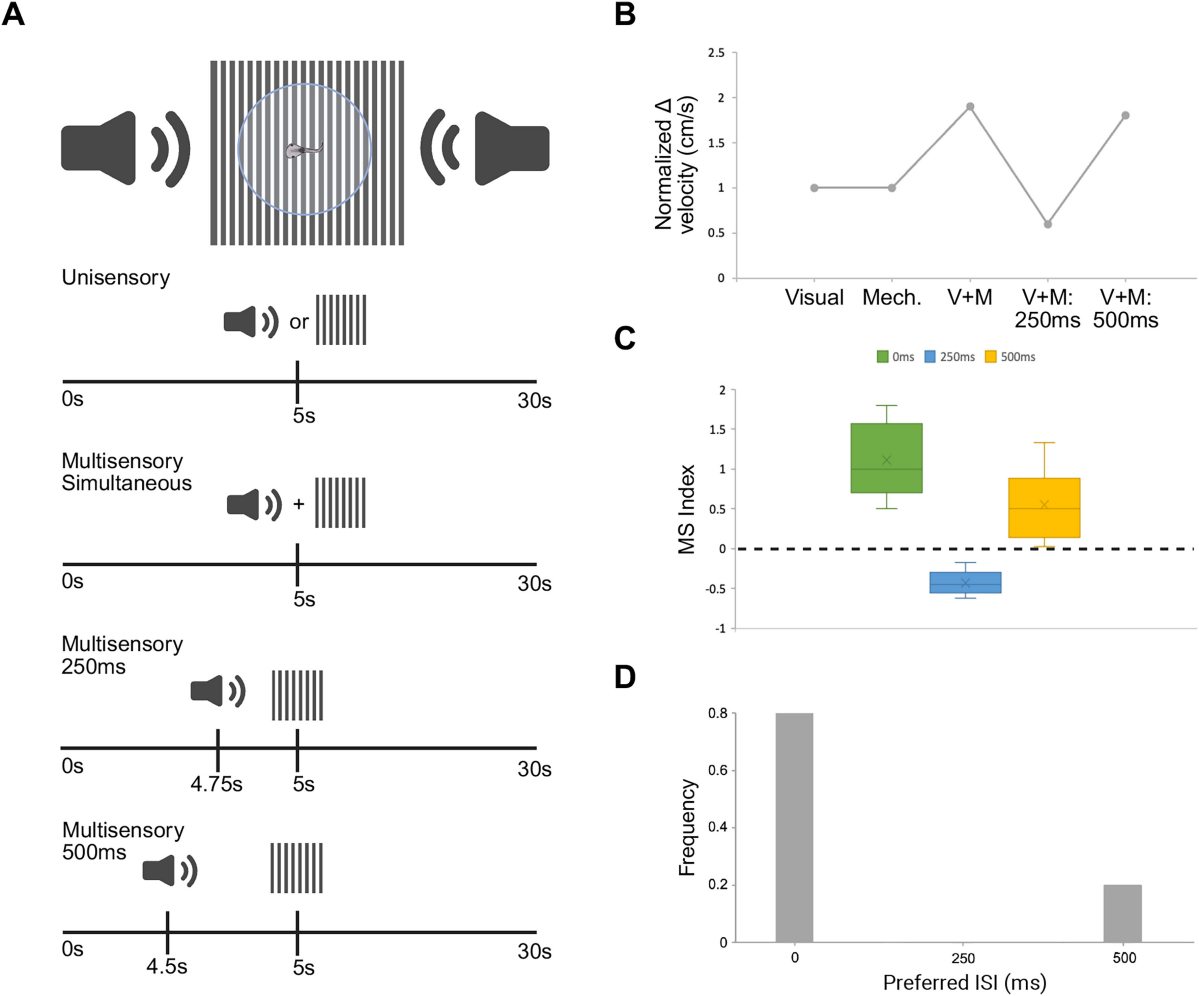
Multisensory integration paradigm in *X. laevis* tadpoles. **(A)** Experimental design. Tadpoles are exposed to subthreshold visual or mechanosensory stimuli, or multisensory stimuli with interstimulus intervals of 0, 250, and 500 ms. **(B)** Normalized change in velocity in control tadpoles in response to visual stimuli, mechanosensory stimuli, or multisensory stimuli at varying ISIs. **(C)** Multisensory (MS) index values for ISIs of 0, 250, and 500 ms. **(D)** Distribution of preferred interstimulus intervals (ISI) across control tadpoles. Adapted from Thompson and Aizenman (2023).

In the MSI paradigm, individual stage 49 tadpoles are placed in a 5 cm dish and exposed to unimodal subthreshold visual or mechanosensory stimuli, or multisensory stimuli with ISIs of 0, 250, and 500 ms (Figure 6A; Thompson and Aizenman, 2023). The subthreshold visual stimuli consist of 25% contrast alternating greyscale stripes at 4 Hz (Truszkowski et al., 2017), while the subthreshold mechanosensory stimuli consist of low-volume clicks that vibrate the dish from both sides without producing a behavioral response in isolation. Tadpole swimming velocity is tracked in real time using a mounted camera, and responses are quantified as the change in velocity averaged over the stimulus window relative to a 1 s prestimulus baseline (Thompson and Aizenman, 2023). These behavioral responses are then used to calculate a multisensory (MS) index, defined as (multisensory–unisensory)/unisensory (Thompson and Aizenman, 2023).

Interestingly, control tadpoles respond most favorably to multisensory stimuli with an ISI of 0 or 500 ms, and tend to slow their swimming at 250 ms ISIs (Figures 6B, C; Thompson and Aizenman, 2023), consistent with the temporal windows for crossmodal inputs to interact reported by electrophysiological recordings, where tectal neurons show a bi-modal distribution of the preferred ISI (Felch et al., 2016). Indeed, most tadpoles responded most strongly to an ISI of 0 ms, while some responded most favorably to an ISI of 500 ms (Figure 6D; Thompson and Aizenman, 2023). Suppression of voltage-gated sodium channel expression during developmental stages 44–46 results in a broadened ISI tuning curve by stage 49, indicating delayed maturation of the tectal circuit (Felch et al., 2016; Thompson and Aizenman, 2023). These results uniquely position the MSI paradigm to detect subtle functional deficits that unimodal assays of visual acuity or acoustic startle responsiveness alone would fail to resolve (Felch et al., 2016; Truszkowski et al., 2017; Thompson and Aizenman, 2023). This would also allow us to detect behavioral deficits in MS integration where the multisensory inputs fail to properly segregate into their respective layers, such as when animals are reared with NMDAR blockers (Hamodi et al., 2016).

### Associative learning

Associative learning is a fundamental cognitive process in which an organism forms a connection between stimuli and events either through prediction or consequences, and modifies its behavior accordingly (Zentall et al., 2014). Associative learning paradigms are powerful tools to study the neural circuits and mechanisms underlying learning and memory and are also commonly used as behavioral readouts of cognitive function in animal models for neurological disorders (Zentall et al., 2014). Numerous associative learning paradigms have been established in a variety of animal models, including rodents (Pearce and Bouton, 2001; Pacchiarini et al., 2017; Sun et al., 2020; Bouton et al., 2021; Takehara-Nishiuchi, 2022; Cassaday et al., 2023; Trent et al., 2025), zebrafish (Blank et al., 2009; Blackiston et al., 2010; Blaser and Vira, 2014; Fernandes et al., 2016; Kenney et al., 2017; Facciol and Gerlai, 2020), *Drosophila* (Boto et al., 2020; Adel and Griffith, 2021), pigs (Gieling et al., 2011), and primates, including humans (Fanselow and Poulos, 2005; Urcelay and Miller, 2014; Pittig et al., 2018; Giovanniello et al., 2023; Towner et al., 2023). Despite the potential usefulness, earlier studies found it challenging to establish reliable associative learning paradigms in tadpoles with the traditional operant and classical conditioning paradigms (Hoyer et al., 1971; Hoyer, 1973; Strickler-Shaw and Taylor, 1991).

#### Characterization and applications of the associative learning paradigm

**FIGURE 7:**
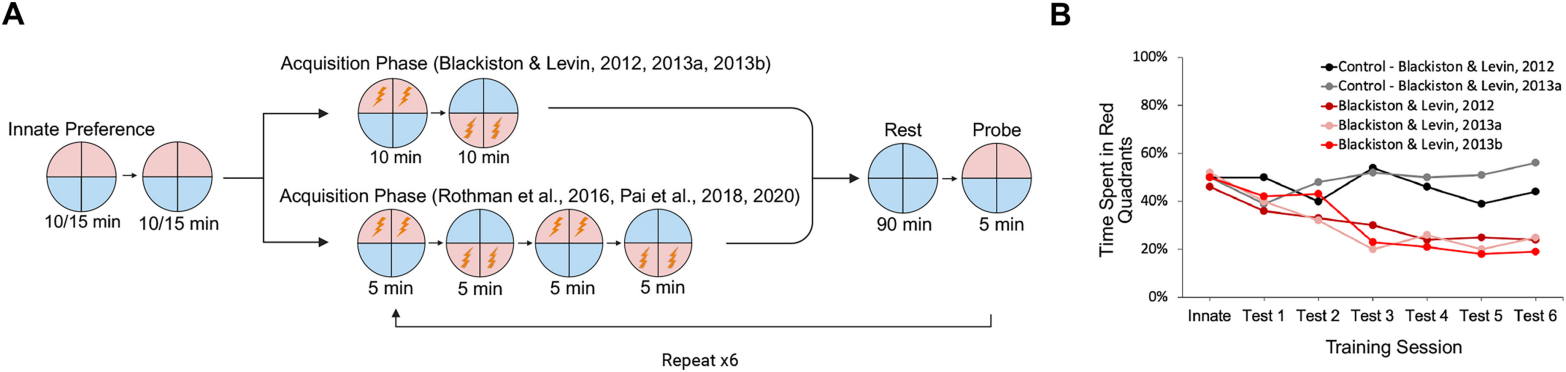
The aversive conditioning paradigm evaluates associative learning in *X. laevis* tadpoles. **(A)** Experimental design consists of an initial innate preference screening followed by an acquisition phase, rest period, and probe session. Animals are trained individually, and the acquisition block is repeated six times. The average time spent on the red half is quantified for each experimental group. Adapted from Blackiston and Levin (2013a), Pai et al. (2018). **(B)** Compilation of the data from different studies showing that stage 48 tadpoles spend less time in the red quadrants following consecutive aversive training sessions. Associative learning plateaus after 3–4 training sessions. Based on Blackiston and Levin (2012, 2013a,2013b), Rothman et al. (2016), Pai et al. (2018, 2020).

A breakthrough came in 2010 when Blackiston et al. (2010) developed an automated closed-loop training paradigm that pairs electric shocks with a specific color, successfully establishing an associative learning paradigm in several aquatic animal models, including zebrafish, planaria, and *X. laevis* tadpoles (Figure 7A). The optimized paradigm in tadpoles consists of four stages: (1) Innate preference. Individual tadpoles are screened for any innate color preference by allowing them to freely swim in a dish with half-red, half-blue illumination for 20–30 min, and any tadpoles that spend > 70% time in one color are excluded from subsequent experimentation. (2) Acquisition phase. Mild electric shocks (1.2 mA, pulsed for 100 ms, followed by 300 ms of no shock) are delivered when tadpoles enter the red half of the dish and are terminated when they swim to the blue half (Blackiston and Levin, 2012; Rothman et al., 2016). (3) Rest period. The entire dish is illuminated with blue light for 90 min. (4) Probe session. Animals are allowed to swim freely in a half-red, half-blue illuminated dish in the absence of electric shock for 5 min, and the percentage of time spent in the red half is calculated.

Animals are individually trained and tested in this aversive conditioning paradigm. During the Innate Preference and the Acquisition Phase, the colors displayed on the dish are inverted at set periods to prevent spatial bias and ensure that stationary tadpoles are punished (Blackiston and Levin, 2012; Rothman et al., 2016). Each training session contains the entire block of acquisition-rest-probe, described above as steps 2–4, and training sessions are repeated consecutively for a total of six training blocks (Rothman et al., 2016), or across two successive days of training, depending on the experiment design (Blackiston and Levin, 2013a). In a six-trial experiment, individual tadpoles were determined to have learned if their averaged time spent in the red half of the dish across the last three probe sessions was below either 30% (Blackiston and Levin, 2013a; Blackiston et al., 2017) or 40% (Pai et al., 2018, 2020). Learning typically plateaus after four training sessions (Figure 7B; Blackiston and Levin, 2012, 2013a,b).

In addition to memory acquisition, memory extinction assays are also useful behavioral paradigms, particularly due to the translational relevance as potential therapeutic strategies for neurodisorders involving maladaptive memory formation (Dunsmoor et al., 2015). In *X. laevis* tadpoles, immediately after associative learning is established during the acquisition phase, extinction trials using neutral probes can be conducted to drive the decay of the learned association (Rothman et al., 2016). During extinction trials, tadpoles swim freely in a half-red, half-blue dish without receiving any punishment. Each extinction block contains two rest periods (90 or 180 min) and two extinction probes, with colors inverted between each probe, and the entire block is repeated 13 times (Rothman et al., 2016). The extinction effects can be modeled as a survival curve, in which the percentage of animals retaining the learned association is plotted for each time point (probe session) — an individual tadpole is deemed to have lost the learned association when the time spent in the red half goes above the extinction threshold of 50% (Rothman et al., 2016). Consistent with prior reports in other animal models, repeated extinction trials were more effective at abolishing the learned association than time passage alone, as evidenced by indistinguishable decay rates with either 90 or 180 min rest periods between extinction probes (Rothman et al., 2016).

The aversive conditioning paradigm has been used to evaluate two aspects of neural function in *X. laevis* tadpoles: (1) cognitive function (i.e., the capacity to learn) and (2) visual function (the ability to distinguish between different wavelengths of light) under both physiological and pathological conditions (Blackiston and Levin, 2013a,b; Rothman et al., 2016; Blackiston et al., 2017; Pai et al., 2018, 2020). Learning can be rescued under certain conditions, including those modeling neurological disorders, indicating the potential usefulness of this paradigm in testing treatment strategies in disease models (Pai et al., 2018, 2020). Establishment of such associative learning paradigms opens avenues for studying the neural pathways and mechanisms underlying learning and memory.

#### Putative neural substrates underlying associative learning

It remains unclear exactly what neural substrates are required for this associative learning paradigm in tadpoles. Interestingly, the capacity for tadpoles to learn in this paradigm seems to be stage-dependent: whereas stage 48 *X. laevis* tadpoles readily learn to avoid the color associated with the shock, stage 47 tadpoles fail to achieve meaningful learning (Rothman et al., 2016). This stage-dependency may lend some insights into the developmental profile of the neural circuitry underlying associative learning. Given the employment of visual stimuli, one important system to consider is the visual system. Although the color-sensitive rods and cones are fully expressed in the retina by stage 47 (Chang and Harris, 1998; Wilhelm and Gábriel, 1999; Bertolesi et al., 2022; Man et al., 2023), the tectal circuit still undergoes experience-dependent plasticity (Ruthazer and Aizenman, 2010), which may play a role in the (in)ability of animals to establish the associative pairing (Rothman et al., 2016). Furthermore, stage 48/49 tadpoles exhibit an innate color preference that is mediated by the tegmentum, suggesting the tegmentum may be involved in color-processing in tadpoles (Hunt et al., 2020). Although the retinotegmental circuit was shown to be relatively stable between stages 46–49 (Udoh et al., 2025), whether the output of the tegmental neurons undergoes developmental changes remains unknown.

Other brain regions outside of the visual-processing circuits may also be involved. For example, the hippocampus and the amygdala are well-known to play critical roles in memory formation and extinction across different species (LeDoux, 2000; Bird and Burgess, 2008). The medial pallium (MP) is the amphibian homolog of the mammalian hippocampal formation and is involved in spatial learning and memory in adult frogs and toads (Bird and Burgess, 2008; Morona and González, 2013; Bingman and Muzio, 2017; Sotelo et al., 2024). In *X. laevis* tadpoles, MP development begins around stage 42 and continues throughout the premetamorphic and prometamorphic stages (Jiménez and Moreno, 2022). Similarly, the amygdala is readily identifiable during premetamorphic stages in the tadpole brain and continues to develop throughout the prometamorphic stages (Morona and González, 2013). However, the functional development of both brain regions in tadpoles remains largely unexplored. Further investigations are warranted to determine which brain areas are required for associative learning in tadpoles and if the maturation of the corresponding circuit underlies the stage-dependency of this paradigm.

#### Methodological considerations for optimization

One limitation of the currently established aversive conditioning paradigm in tadpoles is that it only evaluates the acquisition but not the retention of the learned behavior. As test probes were performed immediately following the acquisition phase, no data is currently available for the long-term maintenance of memory, which significantly limits the use of the paradigm. Furthermore, no reward-based associative learning paradigm has been established in tadpoles thus far. Reward-based social learning paradigms have been successfully established in teleosts such as zebrafish and goldfish (Blane and Holland, 2024). In zebrafish studies, maze designs involving the presence of reward chambers have been used, which tadpole studies may be able to borrow if a proper reward stimulus can be identified (Benvenutti et al., 2021; Reemst et al., 2023). The zebrafish paradigms often make use of the natural attraction of zebrafish to conspecifics, which activates a reward pathway involving increased dopaminergic signaling (Al-Imari and Gerlai, 2008; Fernandes et al., 2016; Reemst et al., 2023). Prior studies suggest that *X. laevis* tadpoles are attracted to water-borne odorants released by siblings, and that sustained exposure to kinship odorants during development is sufficient to increase dopaminergic signaling in the accessory olfactory bulb (AOB) interneurons, a process that further increases attraction to these odorants (Dulcis et al., 2017). This suggests that kinship odorants may serve as a rewarding stimulus to establish reward-based associative learning paradigms in tadpoles.

For labs interested in implementing the aversive conditioning paradigm, a few important factors should be considered for optimal learning in tadpoles. (1) Shock intensity. Tadpoles should be punished with a sufficient but not excessive intensity of current during training. A training current of 1.2 mA, delivered in a homogenous fashion by six electrodes that rotate every 48 ms, appears to be appropriate (Blackiston and Levin, 2012). (2) Feeding schedule. Tadpoles should be well fed both before and during training periods, as learning was absent when tadpoles were starved for 6 h or more (Blackiston and Levin, 2012). In the prolonged absence of food for 3–6 h, tadpoles tend to revert to a “circling” behavior in which they circle the environment, presumably in search of food, regardless of punishment (Blackiston and Levin, 2012). (3) Rest periods. Continuous training periods > 90 min need to be separated by resting periods of at least 90 min for tadpoles to exhibit appreciable learning (Blackiston and Levin, 2012). (4) Rearing conditions. Maintain tadpoles on a strict 12:12 h light/dark cycle and limit tadpole density to no more than 30 tadpoles per 100 × 25 mm dish post-stage 46 (Rothman et al., 2016). (5) The observation that reversing the color assignment from blue-neutral/red-shock to blue-shock/red-neutral did not result in appreciable learning (Rothman et al., 2016) suggests that individual animals may have different innate color preferences between blue and red. In future designs, instead of excluding animals with strong innate color preference, it may be advantageous to design the experiment to associate shock with the preferred color for each animal for a more robust readout of the learning result.

### Free-movement pattern Y-maze

One behavioral assay adapted for *X. tropicalis* tadpoles is the free-movement pattern (FMP) Y-maze paradigm (Ismail et al., 2022). The FMP Y-maze paradigm was initially established in zebrafish and validated in mice and humans as an assessment for working memory, executive function, and cognitive flexibility (Fontana et al., 2019; Cleal et al., 2021). This paradigm differs from the traditional Y-maze by the prolonged exploration period (1 h vs. 5 min) and the lack of either reward or punishment. The readout posits that a cognitively intact animal uses recent spatial memory to guide subsequent movement decisions, producing a non-random, structured pattern of movement. In zebrafish, the structured movement pattern is disrupted by pharmacological interruption of glutamatergic and cholinergic transmission (Fontana et al., 2019; Cleal et al., 2021).

**FIGURE 8:**
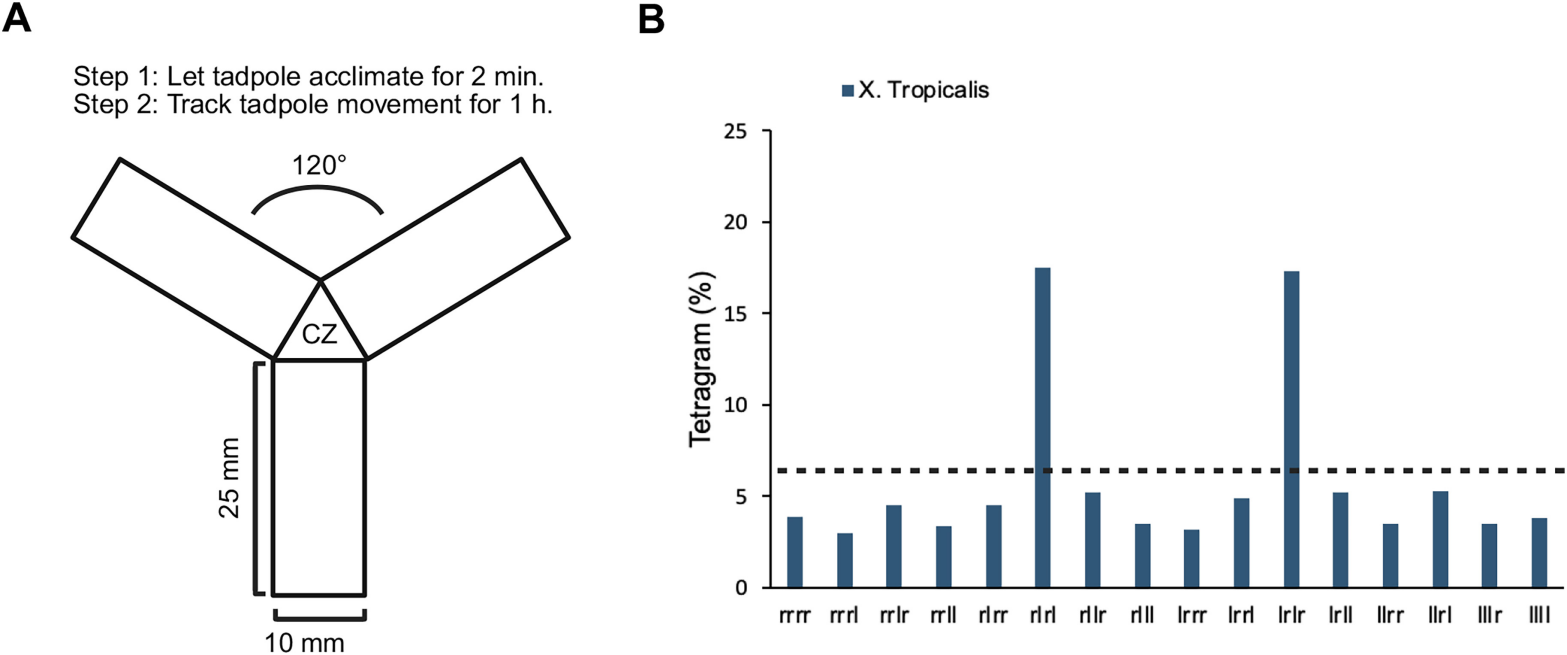
Free-movement pattern Y-maze paradigm using *X. tropicalis* tadpoles. **(A)** The apparatus consists of three identical arms, each measuring 25 mm in length, 10 mm in width, and 10 mm in depth, connected to a 10 × 10 × 10 mm central zone. Tadpoles are placed individually in the maze, allowed to acclimate for 2 min, and then videotaped as they swim freely for 1 h. Sequences of left and right arm entries are analyzed to determine the search strategy. Adapted from Cleal et al. (2021). **(B)** Relative frequency distribution plot for the search strategy of wild-type *X. tropicalis* tadpoles, demonstrating the increased tendency for alternation patterns (LRLR and RLRL). The dashed line indicates the frequency expected by chance (e.g., in the absence of working memory). CZ, central zone. Adapted from Ismail et al. (2022).

In the experimental design using *X. tropicalis*, individual tadpoles at stage 50 are placed into a Y-maze and videotaped for 1 h following 2 min of acclimation (Figure 8A; Ismail et al., 2022). Videos are analyzed *post hoc* to quantify the number of entries into the left (L) and right (R) arms of the Y-maze, in addition to the time of entry and exit from each arm. These entries and exits are converted into binary directional choices (“left” or “right”), and then organized into overlapping sets of four, called “tetragrams” (e.g., LRLR, LLLR), which form the basis of the behavioral analysis. Vertebrates tested on the FMP Y-maze paradigm, including zebrafish, mice, and humans, exhibit a predominant pattern consisting of sequential left-right alternation (LRLR or RLRL), accounting for roughly 25%–30% of all turning decisions (Figure 8B; Cleal et al., 2021; Ismail et al., 2022). Comparison of alternation frequencies between control and experimental groups can therefore be used to interpret working memory and evaluate cognitive deficiencies following molecular or genetic manipulation. Tadpoles with CRISPR-Cas9 editing of the *gria1* gene exhibited significantly fewer alternations in the FMP Y-maze paradigm than wild-type controls, with no significant difference in the total number of turns, suggesting deficits in working memory in the *gria1* CRISPR-edited tadpoles (Ismail et al., 2022).

The FMP Y-maze represents the first and, to date, only behavioral paradigm established for assessing working memory in *X. tropicalis* tadpoles. Therefore, independent replication of the original findings and the extension of the paradigm to *X. laevis* will be critical steps toward establishing the FMP Y-maze design as a broadly applicable tool for cognitive assessment in *Xenopus*. The involvement of glutamatergic signaling via GluA1-containing AMPARs for this behavior in *X. tropicalis* is consistent with findings from rodent and zebrafish models (Ismail et al., 2022). Despite these initial results, however, the underlying circuit-level mechanisms and brain regions involved remain to be characterized. Nevertheless, the availability of a working memory paradigm in *X. tropicalis* opens an exciting avenue for investigating the molecular and genetic underpinnings of higher cognitive functions.

## Social behaviors

### Schooling

It is well-known that many species of aquatic animals, particularly certain types of fish, have an innate tendency to aggregate into groups, termed schooling. *X. laevis* tadpoles also exhibit polarized schooling behavior, characterized by synchronized swimming at similar speeds and in the same directions (Shelton, 1970; Katz et al., 1981; James et al., 2015). Tadpoles start exhibiting schooling behavior soon after they start free swimming (stage 35/36), although the exact stage has not been systematically documented. Tadpole schooling is believed to be advantageous as protection from predators, as well as the production of water currents that may facilitate feeding (Katz et al., 1981). This behavior relies on a combination of visual, mechanosensory, and olfactory cues, making it a useful behavioral assay for the functional development of a variety of sensory systems (Katz et al., 1981; Truszkowski et al., 2017; Liu et al., 2021; Lopez et al., 2021).

#### Characterization and applications of the schooling paradigm

**FIGURE 9:**
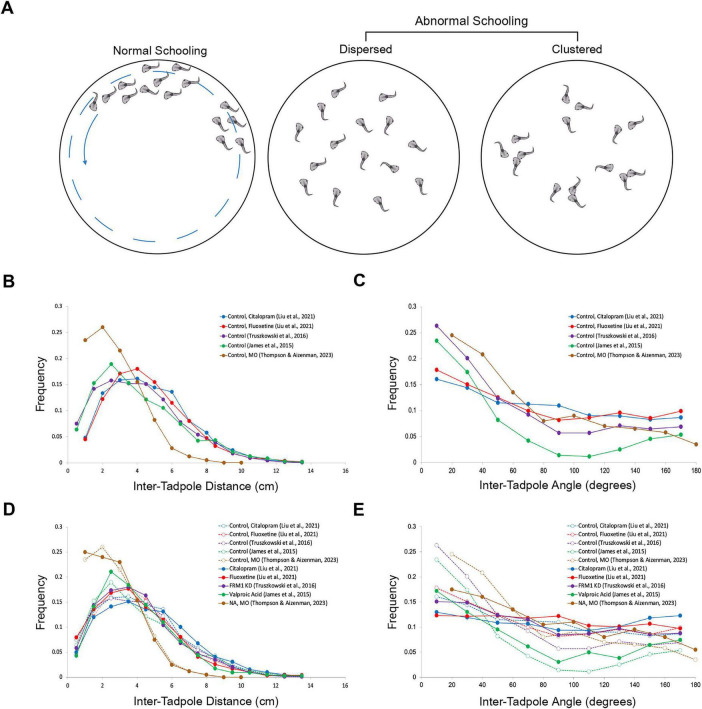
Schooling behavior as a functional readout of *X. laevis* tadpole social interaction and general health. **(A)** Experimental design: 15–30 tadpoles swim freely in a Petri dish while snapshots of tadpole distribution are captured every 5 min for 1 h. Normal schooling is characterized by tadpoles swimming unidirectionally in a congregated group (or a few groups), whereas abnormal schooling is characterized by dispersed swimming, excessive clustering, or tadpoles swimming in different directions. Two examples of abnormal schooling patterns are shown. **(B)** Compilation of data from different studies showing that stage 49 tadpoles tend to swim within 1–5 cm of the nearest neighboring tadpole during normal schooling. **(C)** Compilation of data showing that stage 49 tadpoles tend to swim at 5–50° relative to their nearest neighbor during normal schooling. **(D,E)** Tadpoles exposed to genetic manipulations or pharmacological treatments during early stages of development exhibited disrupted schooling behavior, as measured by inter-tadpole distance and inter-tadpole angle. Controls are presented as dashed, lighter traces; experimental groups are presented as solid, darker traces. Based on Liu et al. (2021), Truszkowski et al. (2016), James et al. (2015), Thompson and Aizenman (2023).

Many key characteristics of *X. laevis* schooling behavior were first described by Katz and colleagues in the early 1980s (Katz et al., 1981). The protocol for quantitative analysis of schooling in tadpoles was established much later (James et al., 2015; Liu et al., 2021; Lopez et al., 2021; Thompson and Aizenman, 2023). Tadpoles are monitored while swimming freely in a group of 15–30 animals within a 17 cm diameter circular dish (Figure 9A). Time-lapse images of the dish are captured every 5 min for a total duration of 1 h (13 images per experiment) to record the position and orientation of the tadpoles. A strong acoustic or vibratory stimulus is presented 2.5 min after each photo is taken to startle the animals. Reformation of schools after the startle stimulus usually takes 30–60 s, ensuring that the following photo captures a new distribution of tadpole positioning and orientation (Lopez et al., 2021).

Schooling behavior is quantified by an automated program that analyzes inter-tadpole distance and inter-tadpole angle. All current data were collected from stage 49 *X. laevis* tadpoles, which tend to swim within 1–5 cm (i.e., 1–2 body lengths) of their nearest neighbor during normal schooling (Figure 9B; Katz et al., 1981; Nieuwkoop and Faber, 1994; James et al., 2015; Truszkowski et al., 2016; Liu et al., 2021; Lopez et al., 2021; Thompson and Aizenman, 2023). Additionally, tadpoles often orient at 5–50° relative to their nearest neighbor, with a strong preference for lower angles, indicating a tendency toward parallel swimming (Figure 9C; James et al., 2015; Truszkowski et al., 2016; Liu et al., 2021; Thompson and Aizenman, 2023). Disruption of schooling can manifest through changes in either the inter-animal distance or angle; however, current data suggest that inter-animal angle seems to be more sensitive to neurodevelopmental deficits caused by pharmacological treatments and genetic manipulations in tadpoles (Figures 9D, E; James et al., 2015; Truszkowski et al., 2016; Liu et al., 2021; Thompson and Aizenman, 2023). In addition, live tracking of individuals and mathematical modeling would allow more sophisticated analyses of the dynamics of school formation and tadpole-tadpole interactions, which would provide further insight into the mechanisms underlying schooling behavior.

#### Putative neural substrates underlying schooling behavior

The neural substrates underlying schooling likely involve multiple sensory systems. Tadpoles tend to swim farther apart and are more likely to orient in parallel in the light than in the dark, indicating that schooling is influenced by visually-guided cues (Katz et al., 1981). The ability of tadpoles to retain schooling in the dark, however, suggests that other sensory systems may be involved as well (Shelton, 1970; Katz et al., 1981; James et al., 2015). The lateral line system is a mechanosensory system that allows tadpoles to detect subtle changes in water movement and pressure gradients generated by nearby individuals (Lum et al., 1982; Simmons et al., 2004). *X. laevis* tadpoles have a well-developed lateral line system that consists of superficial neuromasts, which contain hair cells that respond to displacements caused by water movement (Lum et al., 1982; Lannoo, 1999; Simmons et al., 2004). Consequently, the ablation of neuromasts and damage to hair cells produce significant disruptions in schooling behavior, including altered angles of geometric orientation and increased distances between the nearest neighboring tadpole (Lum et al., 1982; Lannoo, 1999; Simmons et al., 2004). Olfactory cues are also speculated to be involved in schooling behavior as they provide important cues for schooling in many species of fish (Hemmings, 1966), although this has not been directly tested on tadpole schooling. As mentioned above, *X. laevis* tadpoles are capable of recognizing kinship odorants via dopaminergic pathways (Dulcis et al., 2017). Dopaminergic signaling pathways have also been implicated in social behavior in zebrafish, as disruptions to dopaminergic neurotransmission significantly altered social interactions with conspecifics (Facciol and Gerlai, 2020). The functional involvement of olfaction in tadpole schooling behavior and the underlying neural circuits remain to be fully elucidated.

#### Methodological considerations for optimization

For the successful implementation of the schooling paradigm, a few factors should be considered. (1) Control for tadpole density. The tendency for parallel lining increases slightly with increasing tadpole size and density (Katz et al., 1981), suggesting that the same number of tadpoles should be used in each experimental group. In most experimental designs, placing 15–20 wild-type tadpoles in a 17 cm diameter dish is capable of producing schooling behavior (James et al., 2015; Truszkowski et al., 2016; Liu et al., 2021; Lopez et al., 2021), although higher density with groups of 30 tadpoles seemed to produce more robust results (Thompson and Aizenman, 2023). (2) Startle tadpoles with an acoustic or mechanical stimulus in between each photo to encourage redistribution of animals and reformation of new schools (James et al., 2015; Truszkowski et al., 2016; Liu et al., 2021; Lopez et al., 2021; Thompson and Aizenman, 2023). This ensures that successive images do not capture the same distribution, especially if the tadpoles are stationary. (3) Perform experiments at the same time of the day, preferably in the morning, as tadpoles housed in the standard 12:12 h light/dark cycle are found to exhibit the highest amount of swimming activity within 1–3 h into their daylight period (Lopez et al., 2021). (4) Assess general locomotive activity in each group. Schooling behavior is tightly linked to overall swimming activity, and therefore, treatments that increase or decrease movement will likely influence schooling metrics (Lopez et al., 2021). To control for this effect, general swimming activity should be assessed separately for each group to compare baseline swimming speed and motion. (5) Rearing conditions appear to influence the expression of schooling behavior. Tadpoles maintained on a 12:12 h light/dark cycle and reared in sufficiently large volumes (150 mL or so) display robust schooling, whereas tadpoles reared in restricted volumes such as six-well plates fail to develop normal schooling behavior (unpublished observations).

## Concluding remarks

Each of the behavioral paradigms discussed here offers unique functional insights into different aspects of the neural circuits in tadpoles. As behavioral experiments are inherently prone to variability and subjectivity, we suggest several general considerations to improve reproducibility within and across labs. (1) Always include a within-batch and age-matched control. Set a criterion where if controls do not perform the behavior, then exclude that batch. (2) For new experimental implementation, set up ground truth experiments to optimize experimental conditions that induce robust behavioral performance in control animals. (3) Conduct experiments at the same time of the day to avoid circadian variability and stagger light/dark cycles, if needed, to maximize experimental times. Ideally record when tadpoles are most active typically, in the morning. (4) Use a double-blinded experimental design whenever possible.

The growing body of behavioral paradigms in tadpoles and their optimization greatly enriches the toolbox available to researchers for both basic and translational research using *Xenopus* as a model system, opening new avenues for testing functional development under molecular, genetic, and pharmacological constraints. In *X. laevis* tadpoles, these paradigms have proven valuable for linking molecular and cellular mechanisms in visual system development with measurable behavioral output. While the visual system remains the most thoroughly characterized sensory system in tadpoles, it will be of great interest to extend observations from the newly established behavioral paradigms to other sensory modalities and neural pathways. On the other hand, the successful establishment of the FMP Y-maze and innate color preference in *X. tropicalis* tadpoles demonstrates feasibility for the adaptation of other behavioral paradigms. Given the increasing use of *X. tropicalis* as a genetic model for neurological disorders, establishing a behavioral test battery may allow for a comprehensive evaluation of the functional consequences of nervous system development, especially in the case of perturbation. Continuing efforts in establishing new paradigms and optimizing existing ones in tadpoles will greatly benefit researchers as the community strives to bridge cellular and molecular mechanisms with functional output at the circuit level and uncover therapeutic targets in the years to come.
